# A Cross-Chain Solution to Integrating Multiple Blockchains for IoT Data Management

**DOI:** 10.3390/s19092042

**Published:** 2019-05-01

**Authors:** Yiming Jiang, Chenxu Wang, Yawei Wang, Lang Gao

**Affiliations:** 1 School of Software Engineering, Xi’an Jiaotong University, Xi’an 710049, China; magevils@stu.xjtu.edu.cn (Y.J.); wangyawei@stu.xjtu.edu.cn (Y.W.); softlang@stu.xjtu.edu.cn (L.G.); 2 MoE Key Lab of INNS, Xi’an Jiaotong University, Xi’an 710049, China

**Keywords:** blockchain, internet of things, cross chain, data management, access control

## Abstract

With the rapid development of the internet of things (IoT), traditional industries are setting off a massive wave of digitization. In the era of the Internet of Everything, millions of devices and links in IoT pose more significant challenges to data management. Most existing solutions employ centralized systems to control IoT devices, which brings about the privacy and security issues in IoT data management. Recently, blockchain has attracted much attention in the field of IoT due to its decentralization, traceability, and non-tamperability. However, it is non-trivial to apply the current blockchain techniques to IoT due to the lack of scalability and high resource costs. Different blockchain platforms have their particular advantages in the scenario of IoT data management. In this paper, we propose a cross-chain framework to integrate multiple blockchains for efficient and secure IoT data management. Our solution builds an interactive decentralized access model which employs a consortium blockchain as the control station. Other blockchain platforms customized for specific IoT scenarios run as the backbone of all IoT devices. It is equivalent to opening the off-chain channels on the consortium blockchain. Our model merges transactions in these channels for confirmation based on the notary mechanism. Finally, we implement a prototype of the proposed model based on hyperledge Fabric and IOTA Tangle. We evaluate the performance of our method through extensive experiments. The results demonstrate the effectiveness and efficiency of our framework.

## 1. Introduction

The internet of things (IoT) is a networking technology that connects sensors, controllers, and machinery. The IoT technology achieves intelligent management and control of machines through the connection of objects and will inevitably help humanity move toward a more intelligent and convenient future society. With the development of information technology, low-cost information devices have reached households of millions, and the number of devices connected to the Internet is increasing geometrically. According to a report by IBM, the number of connected devices in 2020 is expected to exceed 25 billion units [1]. Traditional IoT models usually consist of a centralized data center that is responsible for collecting and processing data from connected devices. However, this method has drawbacks of high life-cycle costs. Since the maintenance costs of centralized servers are very high, when the number of IoT devices increases to tens of billions, a traditional IoT model may not be capable of meeting the growing demands of the IoT ecosystem.

With the continuous accumulation of unstructured information, most companies do not have practical solutions to the use of their data in order to make profits. The McKinsey research shows that most IoT companies are not yet able to make full use of their IoT data [2]. The first challenge lies in integrating IoT data with other enterprise data in databases and enabling different technologies to work transparently together to fulfil specific goals. Another primary issue is the vast amount of IoT data, and the generating speed is accelerating. However, only a small percentage of these data can be stored permanently [3]. The third problem is the IoT data security. IoT devices generate and process vast amounts of security and safety–critical data as well as privacy–sensitive information, and hence are appealing targets of various attacks [4]. In summary, it is desirable to design a useful and secure solution for IoT data management.

There are already some researches and practices focusing on data solutions for IoT. Jiang et al. [5] proposed a data storage framework for IoT data based on the cloud computing platform. Strohbach et al. [6] developed an integrated big data analytical framework for IoT and smart city applications. However, these models are cloud-centric platforms that the quality of service may be affected by its deteriorated bandwidth and connectivity. Edge-centric IoT-based technologies, such as fog and mist computing, offer distributed and decentralized solutions to resolving the drawbacks of cloud-centric models. Perera et al. [7] reviewed a significant number of IoT solutions in the industrial marketplace. A decentralized consensus system like blockchain is essential to incentivize all participants to share their edge resources [8]. Decentralized consensus systems adopt either blockchain or blockchainless directed acyclic graph technologies to serve as immutable public ledgers for transactions due to its decentralization, traceability, privacy, secure transfer of value and so on. Yeow et al. [8] analyzed the pros and cons of the state-of-the-art decentralized consensus systems and presented several open research issues on decentralized consensus for edge-centric IoT. Dorri et al. [9] proposed a blockchain-based smart home framework and analyzed its security. Their method introduces insignificant overhead relative to its security and privacy gains. Sun et al. [10] discussed how blockchain technology could contribute to smart cities by developing sharing services. Ali et al. [11] designed a network architecture to protect IoT data privacy via blockchains and inter-planetary file system (IPFS). They proposed a decentralized access model for IoT data based on a software stack of blockchains and peer-to-peer data storage mechanisms.

However, since a public blockchain has problems such as lack of scalability and high transaction costs, it is not particularly applicable to IoT scenarios. According to [8], TDAG (Transaction-based Directed Acyclic Graphs) technology (especially IOTA Tangle [12,13]) may be the solution to the vast and growing scale of edge-centric IoT. IOTA is a crypto token specially designed and optimized for the Internet-of-Things. The technology has very low-latency micro-payments in a decentralized M2M/P2P (Machine-to-Machine/Peer-to-Peer) infrastructure. The combination of machines and blockchains transforms these machines into economically independent devices for data exchange. Besides, it also reduces the operating costs of the IoT, solves security threats and protects user privacy.

In this paper, we propose a cross-chain framework to integrate multiple blockchains for efficient and secure IoT data management. Explicitly, we integrate Tangle into the IoT blockchains by building a cross-chain based decentralized access model in the context of data management. In our decentralized access control architecture, we use a consortium blockchain as a control station, while Tangle runs as the backbone of the interconnected IoT devices. We connect the IoT devices to the consortium blockchain through notaries and group the IoT devices for a single use case into sub-Tangles. Our solution provides immutable logs of all IoT data operations, including generation, access and so on. Each sub-Tangle is responsible for maintaining the security logs of the IoT data operations. The sub-Tangle is connected to a more extensive and decentralized consortium blockchain network. The consortium blockchain is responsible for securely recording incoming access to any data and performing access control on these requests. However, it is nontrivial to solve the challenges associated with integrating blockchain to IoT networks.

One of the challenges is the scalability of IoT blockchains. Since each entry on the blockchain requires consensus among all network nodes, the blockchain based on a single architecture do not scale well. To solve this problem, we introduce Tangle using directed acyclic graph (DAG) data structures and decompose the network into multiple smaller sub-Tangles similar to “sidechains”. We then connect these sidechain networks to form a decentralized network. Sub-Tangles are used for different IoT use cases, running on an IoT device cluster, and can be added or removed from the consortium network in a modular fashion. The advantage of implementing sub-Tangles is that nodes in a sub-Tangle do not have to verify transactions that occurred in other sub-Tangles. Users can own and manage one or more sub-Tangles according to different requirements.

Another challenge is the privacy issue of the IoT blockchain. To tackle this issue, we propose an access control model. In order to access a sub-Tangle’s IoT data, the requester must issue an access request as a member of the consortium network. The consortium blockchain prevents any unauthorized access from one cluster of IoT devices to another cluster. Separating the logging responsibilities of sub-Tangles through the consortium blockchain helps to ease the public visibility of blockchain content. However, as far as the architecture is concerned, the members of the consortium network can only access records of IoT data through access requests. However, the log creation events remain private within each sub-Tangle.

We also address other practical problems. To reduce the high multi-agent collaboration cost and prevent individuals from being attacked, we use a consortium blockchain as a control station through smart contracts. To provide secure and trustful data transactions suitable for IoT scenarios, we use Tangle to tag machines to provide cryptographically secure identities and gather data from an object according to its identity. To address the problems of data storage, indexing, and queries for analytics, we use the IPFS [14] and BigchainDB [15] as the decentral storage in an immutable way. To introduce interactive cross-chain verifications between Tangles and the consortium blockchain, we design a particular notary mechanism. To address the privacy and security issues, we design a data access control protocol and new transaction types.

In summary, we make the following contributions:

1. We build a cross-chain interactive decentralized access model in the context of IoT data management.
2. We provide a feasible solution for users to optimize device management and convert their data to cash flow.
3. We design a data access control protocol for IoT data privacy protection in the interaction between sidechains and the consortium blockchain.
4. We implement a prototype of our model and evaluate its performance with extensive experiments. The results demonstrate the effectiveness and efficiency of our model.

The rest of this paper is organized as follows. In Section 2, we compare the advantages of mainstream blockchain systems in the IoT scenarios. In Section 3, we present the proposed architecture of the framework. In Section 4, we describe the implementation of our method and conduct experiments to evaluate the effectiveness and efficiency of the system. In Section 5, we review related work. We conclude this paper in Section 6.

## 2. Blockchains in the IoT Scenario

At present, the problems faced by the internet of things mainly include central server failure, a single point of failure, the infeasibility of fine-grained data sharing, and a lack of privacy. The blockchain techniques have some natural features such as decentralized architecture, fault tolerance, and cryptographic security, to solve these problems.

There are currently a large number of blockchain platforms, including Bitcoin [16], Ethereum [17], Hyperledger-Fabric [18], and IOTA [13]. The mainstream consensus algorithms include proof of work (POW), proof of stake (POS), practice byzantine fault tolerant (PBFT), and IOTA (Tangle). We compare and analyze the functionality, security, and applicability of these mainstream blockchain systems and consensus algorithms in the IoT scenarios. Makhdoom et al. [19] analyzed the difficulties and prospects of combining blockchain with IoT. They believed that the main challenge lies in the fact that IoT devices are growing too fast and transaction concurrency is too strong. The performance of the current blockchain networks is still not up to the requirements of the IoT. Yi et al. [20] conducted preliminary researches on some classic and newly developed consensus algorithms. They suggested that Ethermint implemented by Tendermint is a suitable choice for the blockchain system of the IoT platform. We obtain the four requirements for blockchain systems that are suitable for IoT scenarios, including the ability to provide a heterogeneous network, quick consensus (BFT-based consensus algorithm), no transaction fees, and good scalability.

First, a blockchain platform should provide a heterogeneous network. Nowadays IoT increasingly requires a heterogeneous network that includes multiple sub-networks to handle a wide variety of business logic. For example, the entire smart city IoT can be considered as a heterogeneous network, and sub-networks include smart transportation, smart medical, smart home and so on. These sub-networks have independent businesses and run in parallel. However, they share data to provide a more valuable service. With the development of the internet of things, the blockchain platform is required to provide a heterogeneous network to solve the coordination problem of many sub-networks.

Second, we also believe that a blockchain network with higher throughput, better scalability and without transaction fees is more suitable for IoT scenarios. Fast transaction confirmation is a vital reference factor for the IoT environment, which requires the blockchain to reach consensus without forking. Only BFT-based consensus algorithms satisfy this requirement. Considering that the IoT network needs to process millions of transactions every day, and the number of devices increases persistently, the number of transactions will continue to rise. Transaction fees will bring unaffordable costs for consumers. Hyperledger Fabric has kept this factor optional. However, the blockchain should use strict device authority authentication and well-designed data access control to avoid data storms.

Thirdly, a blockchain system needs to have good scalability. Faced with the rapid growth of IoT devices, the blockchain system needs to handle more and more business. For IoT, such a large number of users, transaction volume, and a large amount of data, or a significant increase in the number of users, the system has put forward higher capacity and scalability requirements, and the single-chain architecture will always encounter software architecture or hardware resources. The system characteristics of the blockchain determine that adding nodes in the blockchain will only enhance the system’s fault tolerance and increase the participants’ credit endorsement without increasing performance.

Table 1 shows the comparisons of several main blockchain platforms. Comprehensive analysis shows that no single blockchain can satisfy the complete requirements of the IoT scenario. However, a combination of Fabric and IOTA is more suitable for the IoT environment.

## 3. Our Solution

### 3.1. The Architecture

**Figure 1:**
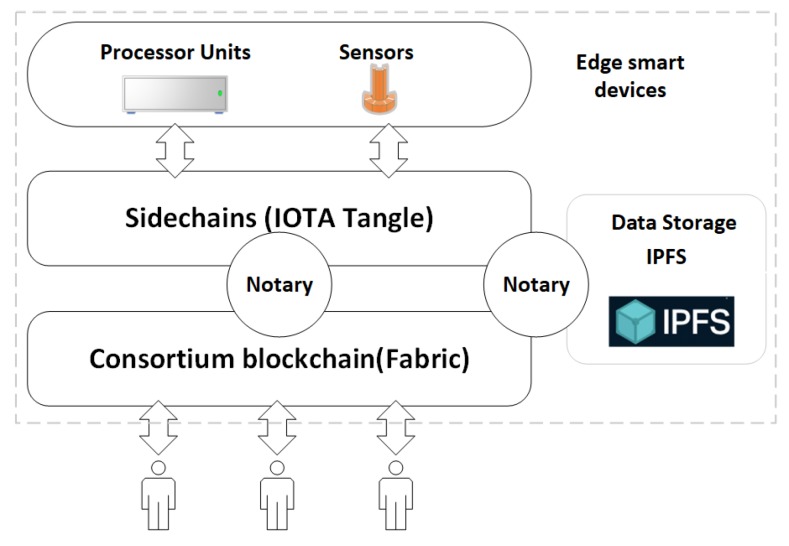
The multi-layer model.

In this part, we present the architecture of our framework from three aspects including the model, blockchain structure, and network topology. We propose a multi-layer model to integrate multiple blockchains for IoT data management. Figure 1 is the multi-layer model of our framework. The model includes three layers. The bottom layer is a consortium blockchain running as a control station. The consortium blockchain connects with multiple sidechains through notaries. The sidechain can be any blockchain systems that are suitable for IoT data management. Edge smart devices such as processor units and sensors form a cluster which is managed by a sidechain. A sidechain can be deployed either on a personal PC for smart home management or clouds for smart factories. A sidechain manages multiple types of devices through the notary mechanism. Another layer of our model is the storage layer. In this paper, we employ the IPFS as the distributed storage system for data storage. Both the consortium blockchain and the sidechains are connected to IPFS through notary nodes.

**Figure 2:**
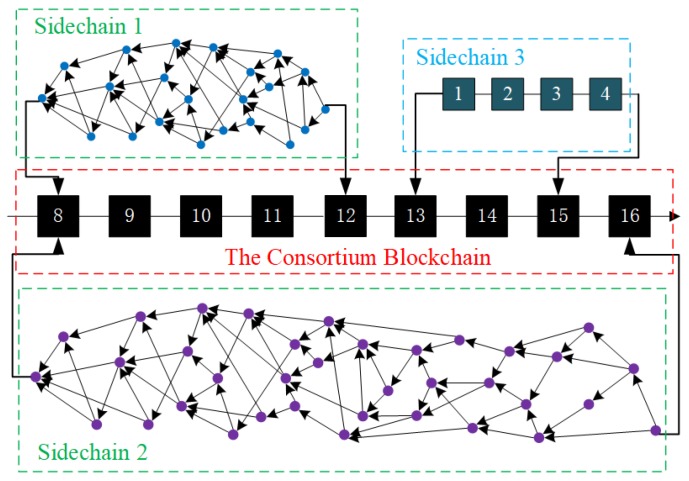
The blockchain structure.

Figure 2 presents the designed blockchain structure. Each side-chain has a specific blockchain structure according to the platform it employs. For example, a sub-Tangle has a DAG structure rather than a continuous chain architecture. A sidechain can be any existing blockchain systems such as IOTA Tangle, Hyperledge Fabric, Ethereum, and Fisco BCOS. The task of each subchain is to record sensor data generation events in its cluster network, while the consortium blockchain is responsible for maintaining a log of successful or failed data access requests from a consortium member to another. Moreover, our model is compatible with direct M2M transactions, a novel micro-payment business model in which IoT devices can initiate financial transactions and can acquire data from other IoT entities to maximize their functions. The model also provides trust mechanisms among devices to prevent great damages to the entire IoT infrastructure caused by the collapses of data centers.

**Figure 3:**
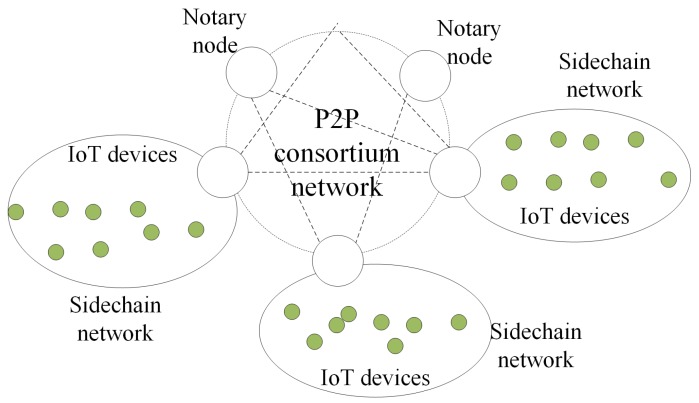
The network topology.

Figure 3 shows the network topology of our architecture. The IoT devices are interconnected with each other in the sidechain network and with the consortium blockchain through notaries. The distributed network architecture of blockchain provides a mechanism to maintain consensus among devices without the need for verification with the centers. Even if one or more nodes are compromised, the data of the entire network system is still reliable and secure.

In this paper, we employ Hyperledge Fabric as the consortium blockchain, and all IoT devices run on Tangle as the backbone of the IoT system. We integrate Tangle into the IoT blockchains and build a cross-chain interactive decentralized IoT data access model. Tangle uses a directed acyclic graph to store the transaction data rather than a chain architecture which adds blocks periodically. “Directed” means that all data is attached in the same direction, and “acyclic” means that the references among data do not form a loop. In traditional blockchain architectures, transactions are grouped in blocks, and each block includes a hash referencing to the previous block. Different from most common blockchain architectures, a node in the Tangle only stores one transaction. In order to attach a transaction in the Tangle, a node needs to verify two previous transactions and add their hashes in the fields of the transaction. A newly added transaction is confirmed when it is referenced by a milestone transaction directly or indirectly. A milestone transaction is generated by Compass which is a coordinator application that is used to protect Tangle against attacks. Indirect reference means the transaction is referenced by an ordinary transaction which has already been confirmed. Through DAG, Tangle achieves higher transaction throughput (through parallel verification) without transaction fees. As Tangle continues to develop, more and more participants will initiate transactions, the entire system will become more and more secure and fast, confirmation time will be shortened, and transactions will be completed faster and faster.

### 3.2. Consensus and Notary Mechanism

The consensus is a critical challenge to integrate multiple blockchains. In this paper, we propose a notary mechanism to reach consensus between a side-chain and the consortium blockchain.

#### 3.2.1. Notaries in the Consortium Blockchain

**Figure 4:**
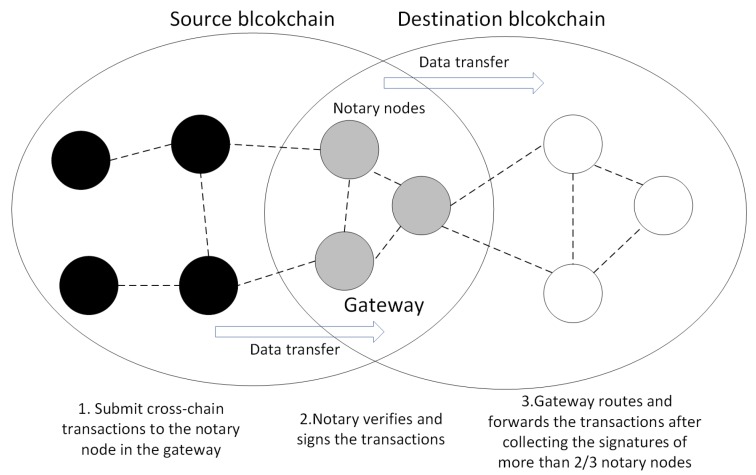
Notary network as a gateway.

We reinterpret the consensus protocol as a verification protocol in the context of cross-chain interaction. The verification protocol will verify that the transaction is confirmed in the source chain and whether the transmitted data has been distorted. Notary nodes between the consortium blockchain and the sidechains form a network which acts as a gateway of different blockchain systems. The cross-chain gateway is responsible for reading the information on both chains and routing cross-chain transactions. The notary network confirms each cross-chain transaction by the voting mechanism, and the transaction is confirmed when the signatures of more than 2/3 notaries in the network are collected. Figure 4 illustrates the data flow between two blockchain systems through the notary network. That is, a group of nodes participate as a relatively independent role which verifies transactions from both parties. Only transactions verified by notary nodes will be transmitted to the destination blockchain through the gateway.

The consortium network has one or more notary services, which provide transaction sequencing and time-stamping services, thereby transforming miner roles abstracted by other systems into a pluggable component. Notaries provide ordering services for consensus in the consortium blockchain. The notary consists of multiple participants/peers who do not trust each other, using a standard consensus algorithm such as proof of work (PoW). The notary is identified and signed by a composite public key, which follows the cryptographic criteria of the general ledger. The byzantine fault tolerance (BFT) algorithm is generally used for notary services. When it is sufficient to ensure that the protocol is strictly followed, high-performance algorithms (such as Raft) can be adopted, e.g., a private blockchain.

Notaries accept the transactions submitted to them, either return the signature of the transaction or return a rejection error if double spending has occurred. Once all other necessary signatures have been gathered, the transaction flow can be ended by calling a trigger. We remove the notaries who no longer provide services. We can further increase the scalability of the network by running notaries online in parallel. The regulatory constraints on data dissemination may be the responsibility of specific notary with jurisdiction.

#### 3.2.2. Notaries in Sidechains

In this part, we take the Tangle as an example to show the design of notaries for sidechain systems. Unlike conventional blockchain technology, Tangle does not have a concept of time series that each block is connected to form a chain, and the situation that each block in the blockchain contains some transactions also does not exist in Tangle. In Tangle, each transaction is directly connected with other transactions (so-called connections, that is, verifying other transactions) and can occur in parallel. Tangle’s consensus mechanism is that the user must randomly pick two tips from the Tangle (the tip is the unconfirmed transaction) and verify the two tips in order to add a new transaction to the Tangle. The so-called verification means that the user needs to check the signatures of the tips and make sure that the selected tips do not conflict with any previous transactions (either directly or indirectly). If the selected tips are legal, the user refers to it and adds the new transaction.

An IoT network of a single use case (a sidechain network) consists of a devices cluster and the notary nodes responsible for maintaining the sub-Tangle. Each IoT device has a unique public and private key that is used to send encrypted sensor data to the notary nodes. The notary nodes record any data received and encrypt it using the authorization key. The notary nodes are responsible for merging the sub-Tangle into the main Tangle network as the full node of the Tangle network. Therefore, notaries are required to have higher computational power and storage spaces. In practice, we employ edge computing techniques to build a sidechain network.

**Figure 5:**
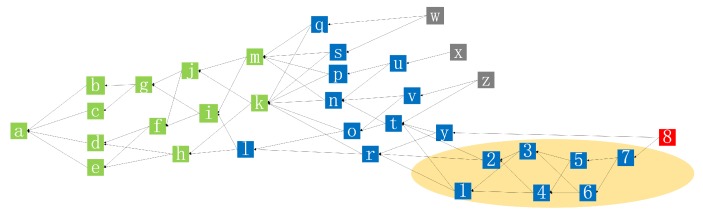
Merging of a sub-Tangle.

Due to the partition fault tolerance of Tangle, a new structure of Tangle can be generated outside the main Tangle network. The DAG structure outside the main network can be viewed as a sub-Tangle and can later be merged back into the main network. Each sub-Tangle confirmed by the main network are equivalent to a side chain of the main Tangle. To merge the sub-Tangles, we firstly create transactions according to the Tangle consensus on the IoT device cluster and connect them to form a sub-Tangle. As shown in Figure 5, transactions 1 and 2 are created by the notary node who connects to the device clusters that need to merge the sub-Tangle. Both transactions are connected to the tips of the main Tangle that are the last known. We build the main Tangle which is a federation network via compass provided by IOTA. Compass is a coordinator application that can be used to protect Tangle against attacks by sending honest and zero-value transactions, and any transaction which is referenced by a milestone transaction is considered to be confirmed in the network. In our implementation, the notary plays the role of Compass. If any transactions in a sub-Tangle conflict with the main Tangle, they will be rejected by the notaries and the cluster of the sub-Tangle is considered dishonest. The verification is performed before merging the sub-Tangle, and subsequent transactions are constantly attached to the tails as usual. Since all transactions and zero-balanced addresses are deleted after Snapshot, to prevent it from happening, we consider adding a node that can store transactions permanently. Once there is a commit to the main Tangle, the sub-Tangle will be finalized through transaction 8 created by notary nodes. It will merge the sub-Tangle with the tip of the current main Tangle. Subsequently, transaction 8 becomes a legitimate tip and is available for selection and verification of subsequent transactions. The next transaction connected to transaction 8 will include all transactions on the sub-Tangle on their verification path. Only when sub-Tangle transactions are added to the main Tangle like other transactions, will these transactions be fully confirmed. If any transactions in the sub-Tangle conflict with the main Tangle, transactions 1 to 8 will not be confirmed. The Tangle network’s notary maintains all these operations. It is equivalent to opening an off-chain channel of the DAG Tangle structure on the central consortium blockchain. Transactions in these channels form the sub-Tangle which are then merged into the main Tangle for confirmation. The merging of sub-Tangle is shown in Figure 5.

When merging Tangle networks, both parties from different IoT device clusters must believe that the other notary has never signed double spending. When a sub-Tangle is merged into the main Tangle, it solely relies on incentives to provide this assurance under the consortium network. If more evidence is needed, an independent notary can run on secure hardware like Intel SGX [21] which is enabled for audit logging. User may reattach their transactions when the transaction has been pending for a long time. Reattachment means to create a new one and attach it to a different part of the Tangle. Promoting is often more effective than reattachment especially in our network maintained by a Compass coordinator. Users need to create and send a zero-value transaction that references both their transactions and the latest milestone. In our implementation, we do not consider the reattachment of transactions.

We also use the Oracle service when one blockchain needs to read data from another blockchain. The Oracle service can also provide real-world data outside the blockchain. Oracle is defined as a trusted network service that signs a transaction that contains a statement about the outside world of the ledger. It is assumed that the statement must be true and can be verified easily. Notary nodes maintain the Oracle service. The way to implement Oracle is to have the notary nodes sign a small data structure, and then embed the data structure in the transaction. Then the Oracle signs the entire transaction and provides a timestamp. The signature process is completed when the signature of more than 2/3 notaries in the network is collected.

### 3.3. Data Storage System

**Figure 6:**
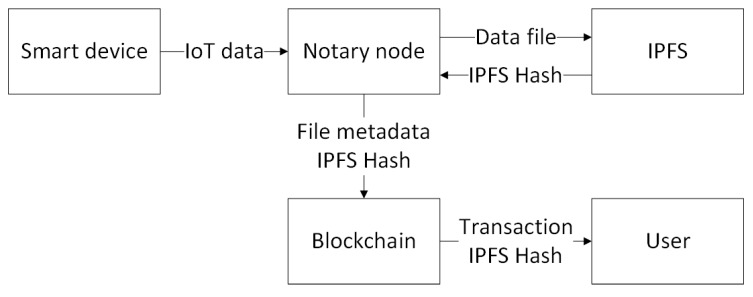
A flowchart of the data storage process in our model.

Storage on the blockchain is costly. Meanwhile, the amounts of IoT data increase continuously. Therefore, it is a great challenge to design a storage system for IoT data. In our solution, we employ IPFS to solve this problem. IPFS is short for inter-planetary file system, which is a distributed peer-to-peer file system. We employ the IPFS to store the blobs with directory and file hierarchies as a file system. Figure 6 presents a flowchart of the data storage process in our model. When adding a file to the IPFS, the file is divided into blocks, and all of the blocks are given a unique fingerprint called a cryptographic hash. Then, IPFS removes duplications across the network. Each network node in IPFS stores only content it is interested in, and some indexing information that helps figure out who is storing what. When looking up files, we are asking the network to find nodes storing the content behind a unique hash [22]. In our solution, IoT data is grouped and stored in IPFS, while the blockchain stores the hash of the IPFS file containing the IoT data. These hashes can then be used to find the actual location of the file.

The following problem is the access control of the IPFS file. Anyone can access the shared files if they have a hash of them. We use the cryptographic public and private keys to solve this problem. When uploading and accessing files on IPFS, we can grant access to specific users by encrypting the data with the public key of the recipient. We employ blockchain programmability and PKI for secure access control. We build product databases of IoT devices based on the blockchain technology provided by BigChainDB. The databases record the complete history information of IoT devices, which are used to verify provenance, authenticity, and ownership. We mark the physical machine’s mac identity on the Tangle. We use the BigchainDB access token for access management to give full control of the data to the owner of the devices.

### 3.4. Access Control Strategy

**Figure 7:**
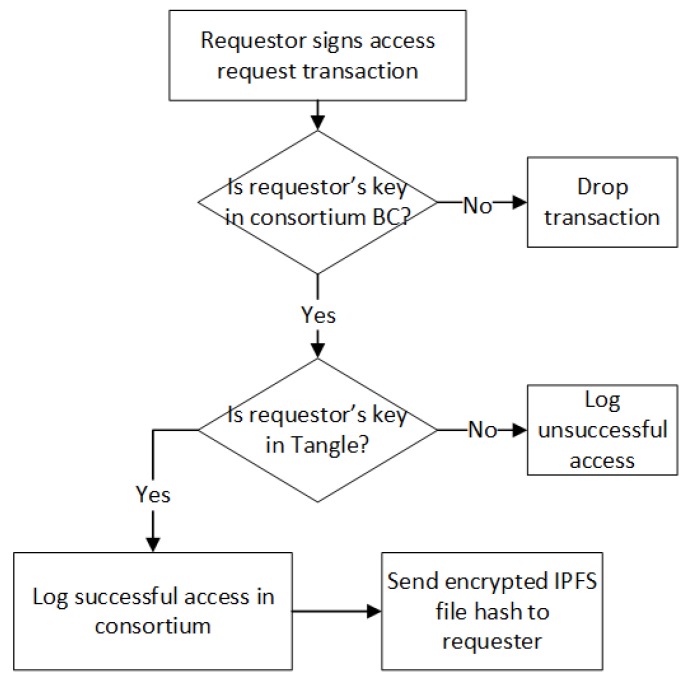
Flowchart of access control.

Smart contracts bring programmability to blockchains, and we can use smart contracts to facilitate transaction execution while complying with contractual terms written in code. The node signs the transaction and sends it to the smart contract to invoke specific functions. In our model, we deployed smart contracts for access control. We implement the required terms and conditions of financial transactions in smart contracts. We deploy smart contracts on the consortium blockchain to implement the access control policies. We also use smart contracts to store the smart devices’ public keys and the hashes of the IPFS files which contain the data of the smart devices. We also store the public key of the requester with access rights in the consortium network. Smart contracts ensure that only authorized smart device’s data can communicate with the notary and perform access control on incoming access request transactions. The flowchart of our access control model is presented in Figure 7.

The decentralized consortium network is formed by the interconnection of the notary nodes in each sub-angle. The consortium blockchain runs on the whole network. Smart contracts are deployed on the consortium blockchain to store the public keys of the smart devices which are authorized to access the IoT data. When new IoT devices are added to the network, each of them will be assigned a public key which is stored in the Tangle and data storage system. The sub-Tangle is responsible for storing the IoT data and recording all local IoT events securely. The consortium blockchain is responsible for checking and allowing external requesters to access the IoT data. The access control policies in smart contracts limit the requester’s rights to access only the data of devices within a particular IoT device cluster. The notary node identifies any unauthorized device access to the sub-Tangle and blocks all incoming data from unauthorized devices. We maintain a list of authorized smart devices in the notary node. This mechanism prevents unauthorized devices from using fake spam data to attack the notary node. Once the data is generated, the authorized smart devices encrypt the data and send them to the notary node. After receiving data from the authorizing device, the notary node records the data generation transaction and attaches it to the last known tip of the main Tangle. The notary node then uploads the IPFS file and stores the corresponding hash value.

**Figure 8:**
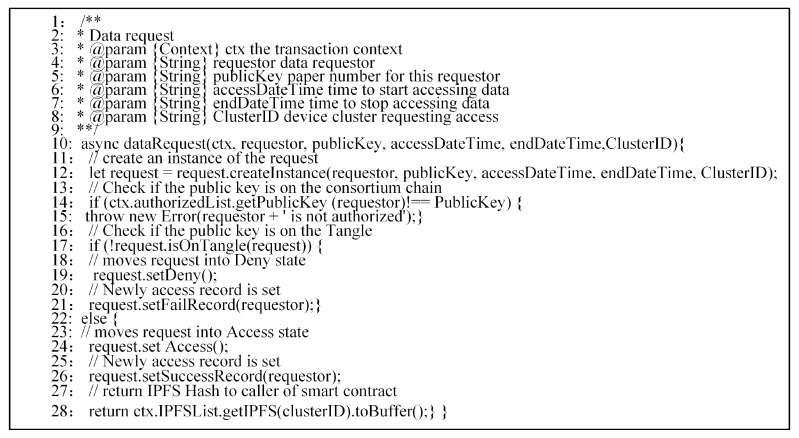
The chaincode of access control data request.

To access the IoT data, the requester has to join the consortium blockchain network as a member and run a client as a peer. In the case of IoT data sharing or trade, the requester’s public key and the requester’s access time is added to sub-Tangle by the owner of an IoT device cluster. The notary node then adds the public key of the new requester to the list of authorized members in the consortium blockchain. At this point, the requester uses its private key to sign the access request transaction and then checks to see if its public key is in the consortium blockchain and sub-Tangle. If so, the hash of the encrypted IPFS file is sent to the requester. The requester downloads the encrypted IPFS file according to the received hash value of the IPFS file and decrypts it using its private key. Thus, it accesses the file containing the IoT data. This policy ensures that the requester can issue access requests at the consortium level only when the IoT device cluster owner provides guarantees. We also employ smart contracts on the consortium blockchain to prevent requesters from launching denial of service (DoS) attacks by flooding the services with many unauthorized requests. If the number of consecutive unsuccessful requests is greater than a predefined threshold, the smart contract will delete the relevant public keys of the requester from the authorized list for punishment. Figure 8 presents a brief implementation of the access control policy since our smart contract chaincode contains about 200 lines of codes.

## 4. Experiments

### 4.1. Implementation

**Figure 9:**
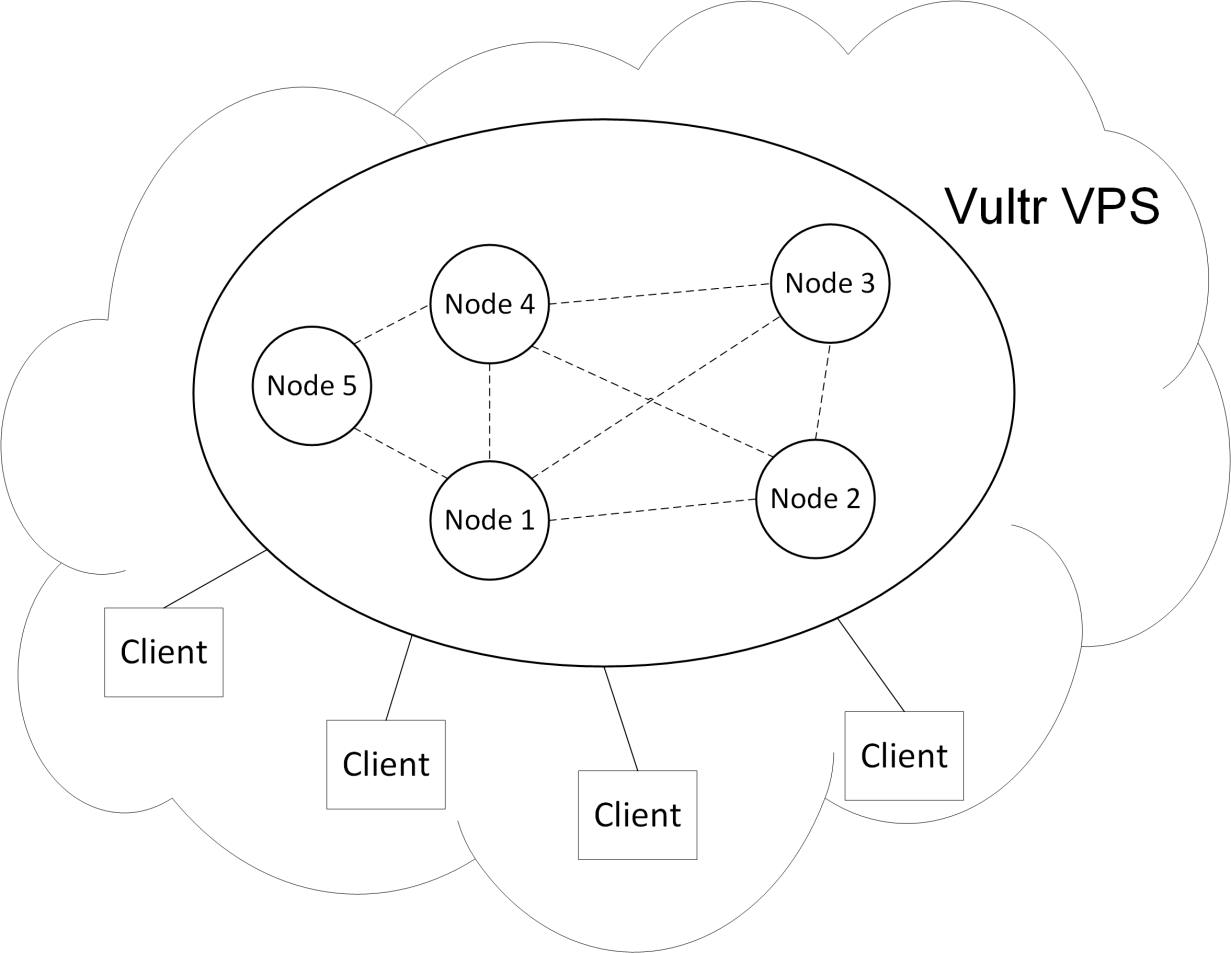
Experimental setups.

We implemented a prototype of the proposed model. We build a consortium blockchain based on the Hyperledger Fabric [23]. Hyperledger Fabric is a business blockchain development platform which uses the BFT algorithm to reach consensus. We built a private Tangle based on the IOTA architecture. Figure 9 illustrates the experimental setups. We deployed five nodes on the Vultr VPS [24] with Ubuntu 16.04 xenial. Nodes 1, 2, 3, and 4 served as typical peers of the Hyperledger Fabric and Node 5 served as the orderer node of the Hyperledger Fabric. Nodes 1, 2, 3, and 4 also run full deployments of IOTA. We implemented the notary gateway network on Nodes 1, 2, 3, and 4, which also connect to the public IPFS service. Every node received data from multiple Hyperlegder Fabric clients that can interact with the blockchain network.

In the following experiments, we evaluate the performance of the system by examining the metrics of throughput, latency and resource consumption such as memory, CPU, and network traffic overhead.

### 4.2. Throughput and Latency

We tested the throughput and latency for different transaction send rates under different configuration environment of the node. In this experiment, we tested the throughput and latency of our P2P consortium network by varying the send rates. We generated and sent 5000 transactions by the APIs provided by Hyperledger Fabric in each test. All transactions are queries to access the data on Tangle via Hyperledger Fabric. The chaincode (smart contract) in Hyperledger Fabric was ran as a container. The results are presented from Table 2, Table 3, Table 4 and Table 5. Tps means transactions per second.

From the results, the throughput generally increases as the send rate increases. Moreover, as memory and the number of CPU cores increases, the throughput is closer to the default send rate, the latency of transaction processing also gradually decreases. Especially when the number of CPU cores increases, the performance improvement is more prominent.

### 4.3. Resource Consumption

**Figure 10:**
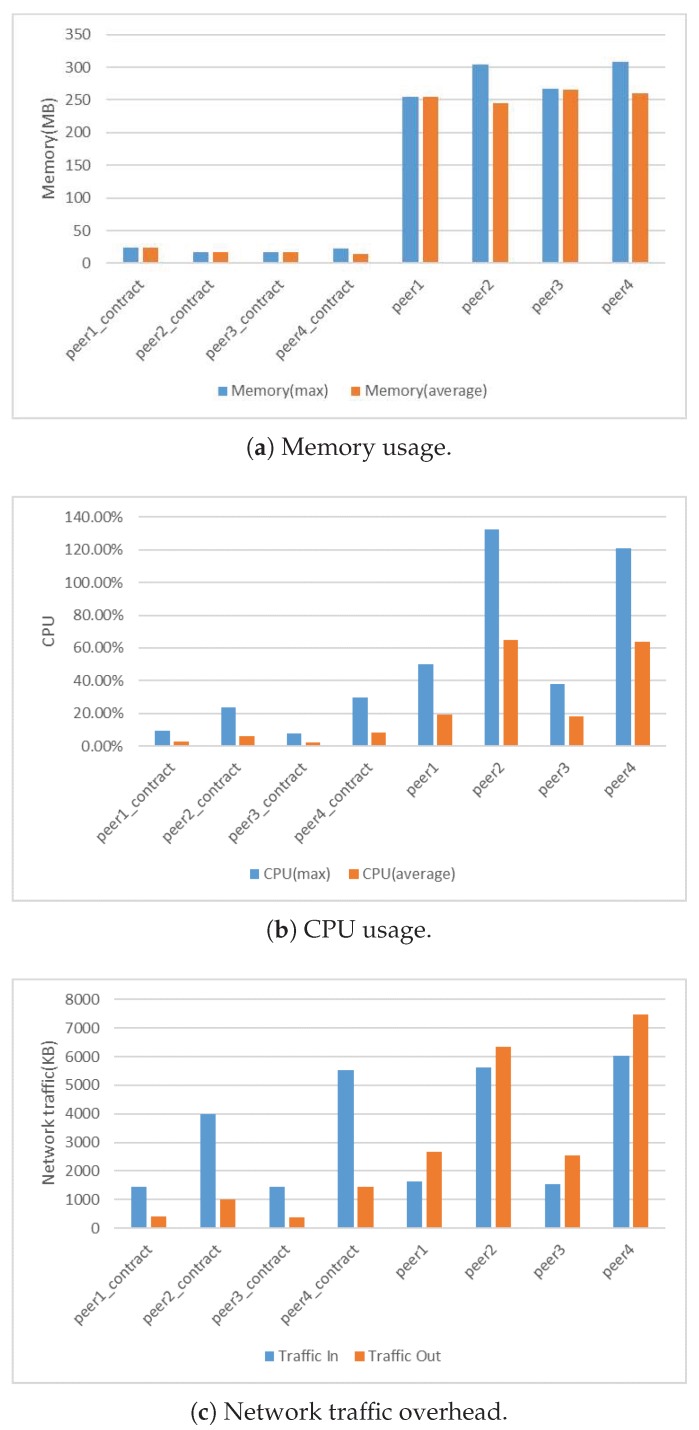
Resource consumption.

We also tested the memory, CPU and network traffic usage of the system. We chose results in the 4G memory and four cores configuration. Figure 10 presents the results. We compared the processing resource consumption by comparing the average memory and CPU usage. The results presented in Figure 10a,b show that a Fabric peer required at least 300 MB of memory, and an average of 40% CPU usage. From the perspective of memory and CPU usage, Hyperledger Fabric was not suitable for deployment in IoT devices with limited resources compared to Tangle light nodes. In blockchain applications, network nodes communicate with each other to reach consensus. In this experiment, we also measured the traffic overhead of the consortium network. We evaluated the traffic overhead of our blockchain network by monitoring the input and output traffic of the peer container of the Fabric network. Network traffic overhead mainly includes transactions and data disseminated through the network. Figure 10c shows that the BFT consensus has huge network traffic overhead. In order to reduce the traffic flowing into the blockchain network, we can use fog nodes as notary nodes to process IoT data in practice, which can significantly reduce the network congestion. Besides, we also compared the peak memory usage of a specific process of different Tangle node and clients. Table 6 displays the results.

From the results, because the contract installed on the peer in the Fabric started a separate runtime environment in the form of a container, this container was only responsible for providing the contract with the running environment and the processing of contract calls, so the overall resource consumption was relatively low compared to the peers. During the query, the peer’s out-traffic was significantly higher than the in-traffic. It was because the query transaction queried the ledger on the peers by calling the contract and the peers send query result back to the requester.

### 4.4. Deployment Considerations and Analysis

From the experiments above, we can see that due to the memory occupied by the Tangle full nodes and the consortium blockchain, the memory required for the notary node was relatively large, and the memory occupied by the light node client in the edge device is tiny compared to notaries. Besides, our notary node’s CPU usage in different scenarios during a day remained almost the same. Therefore notary nodes can be deployed on the servers of each member of the consortium. Through our use of the consortium network, we found that network traffic had increased significantly in the case of large transaction volumes. The results of network traffic overhead show that the BFT consensus had huge network traffic overhead. In order to reduce the traffic flowing into the blockchain network, we can use fog nodes as notary nodes to process IoT data, which can significantly reduce the network congestion. The throughput of our network was mainly affected by the consortium blockchain. When the transaction request per second reaches the processing limit of the consortium blockchain, the throughput of our consortium network becomes stable. However, the throughput of Tangle still increased as the transaction rate increased. It was because transactions on the sub-Tangle can be verified in parallel on the Tangle, which enhanced the processing performance of the system.

## 5. Related Work

The combination of blockchain and the Internet of Things has attracted the most attention recently. Due to the lack of an IoT-centric consensus protocol, the existing blockchain system cannot meet the requirements of the Internet of Things in terms of transaction delay and scalability. Many approaches have been proposed to address these problems. Sagirlar et al. [25] designed a hybrid blockchain architecture for IoT which is named as hybrid-IoT. In hybrid-IoT, IoT devices form subgroups which use Proof of Work consensus algorithms. We refer the subgroup as POW sub-blockchains. The POW sub-blockchains employ a BFT interconnection framework, such as Polkadot or Cosmos. Li et al. [26] used the blockchain system to design an identity authentication mechanism and a data protection mechanism for IoT devices. Compared with their proposed system, our advantage is that IoT devices can customize their access control and have more autonomy. Alphand et al. [27] proposed IoTChain, which is a combination of OSCAR architecture and ACE authorization framework. IoTChain provides an end-to-end solution for securely authorized access to IoT resources. In the system they propose, the data is stored by the server, and we use IPFS to store the data. Our models have lower storage costs and greater data privacy, preventing problems such as single points of failure on the server. Ouaddah et al. [28] proposed FairAccess as a new decentralized pseudonymous and privacy-preserving authorization management framework. The framework leverages the consistency of blockchain technologies to manage access control. However, the solution has not yet been implemented. Oscar Novo [29] developed a fully distributed access control system for IoT based on blockchain technology. However, the system suffers high transaction latency since underlying blockchain based on the Ethereum. Reilly et al. [30] developed an integrity-first communication protocol for IoT based on the Ethereum blockchain. They implemented light clients for the resources limited IoT devices by reducing the code related to the miner and avoiding to store data on the blockchain. However, they do not solve the transaction latency problem. Puthal et al. [31] proposed a new consensus algorithm named PoAh (Proof-of-Authentication) for Scalable Blockchain in Resource-Constrained Distributed Systems.

There have been many techniques taking the advantages of blockchain in different ways to improve the IoT services, although the combination of blockchain and IoT is relatively new. Blockchain can benefit IoT techniques in many scenarios. For example, sensors attached to many IoT devices can be traced through the traceability of blockchain, which provides useful information about the distributions of the devices. Analogously, the blockchain technology also enriches the sensed data provided by many IoT applications.

Ethereum [17] is one of the most popular platforms for IoT blockchain applications. It has more features than Bitcoin. Besides, Ethereum provides the function of smart contracts which significantly boosts its applications in IoT scenarios. Slock.it [32] proposes a blockchain framework to address security, identity, coordination, and privacy over billions of IoT devices. It aims to build a sharing economy where each IoT asset can be rented securely and quickly without the need for any authority [32]. MyBit [33] aims to build an ecosystem of data-sharing services. People can share the revenues of their owned IoT assets (e.g., drones, cars, smart home, and so on.). It employs Ethereum smart contracts to fulfill the goal. The project of Atonomi [34] is built on the Ethereum network. The goal of Atonomi is to provide a universal trust environment for billions of IoT devices through its device identity registration service and reputation service. IoTex [35] is a decentralized network for the Internet of things powered by a privacy-centric blockchain. The root chain manages several separate blockchains or sub-chains which are connected to similar IoT devices. Waltchain [36], similar to IoTex in architecture, is committed to combining the blockchain with RFID (Radio Frequency Identification) in order to solve the problem of product traceability.

The autonomous decentralized peer-to-peer telemetry (ADEPT) [37] IoT system was jointly created by IBM and Samsung and uses blockchain technology to create a decentralized IoT. The full name of ADEPT is “autonomous decentralized peer-to-peer telemetry”, which aims to provide optimal security for transactions. The system is based on three protocols: Blockchain, BitTorrent, and TeleHash. ADEPT employs Ethereum [17] which is a smart contract-based system, and ADEPT can enable devices connected to it to communicate securely and efficiently with each other and implement complex business logic. ADEPT is a user-centric complete IoT platform, similar to IOTA Tangle. However, IOTA does not use a blockchain. Instead, it uses a distributed database model called Tangle (DAG), which is technically superior compared to a blockchain in terms of scalability and the feasibility of micro-payments.

Unlike the use of Ethereum, Innogy [38] combines IPFS, IPDB, and IOTA in supply chain/IoT applications. IPFS is used for file systems and Blob storage. IPDB (using BigchainDB) is used for metadata storage and query queues. Innogy utilizes physical objects through the supply chain and provides the “trust-to-data”. These datasets are tracked automatically and verified through IOTA disputes in the supply chain. Innogy aims to create digital twin [39]. A digital twin is a digital representation of a physical, biological or digital object. The Innogy Digital Twin project Twin of Things seeks to give every physical object a story. Moreover, the first practical application of a digital twin with IOTA is CarPass for vehicles telematics data. The CarPass solution securely captures telematics data and stores them immutable in the digital twin for private passenger, fleet or commercial vehicles.

IOTA has proved scalable due to the particular DAG structure of transactions. While running smart contracts on IOTA is a challenge, the combination of Hyperledger Fabric and IOTA Tangle can well address this problem. We integrate Tangle into the blockchains, and our cross-chain interactive decentralized IoT access model considers better data access privacy. We use a consortium blockchain running in a control station, while Tangle as the backbone of the IoT system runs on all the devices, this architecture takes full advantage of the features of blockchain and Tangle. Different from existing work, our solution uses the BFT consensus algorithm. The throughput of our proposal is acceptable considering the overhead of cross-chain interaction. Besides, due to the scalability and the nature of parallel verification of Tangle, as the number of participant nodes increases, Tangle can lead to higher throughput and better system performance.

## 6. Conclusions

In this paper, we propose a cross-chain framework to integrate multiple blockchains in the context of IoT data management. In order to increase the scalability of the IoT blockchain, we design a notary mechanism to maintain a cross-chain network and apply IPFS and BigchainDB on the notary node to solve problems of data storage and device tagging. In order to address the privacy issues of the IoT blockchain, we propose a data access control model and design a particular transaction type to provide fine-grained access control of data to different chains and nodes. Finally, we implement a prototype of the model based on Tangle and Fabric blockchains. We conduct experiments to evaluate the efficiency of our framework. The experimental results show that our framework is suitable for the management of IoT devices with less resource and is convenient to deploy in the IoT scenarios among multiple consortia. Besides, our model is more efficient than the general blockchain structure. Due to the centralization of the Tangle based on the coordinator compass deployment, we need to sacrifice some decentralization compared with the current public blockchain architectures. The experiment is currently based on software simulations and has not been deployed on the real IoT devices. In the future, we will add real sensor devices, test real M2M transactions, and conduct further experimental analysis and optimization of resource consumption for IoT deployment. Besides, the current privacy access control scheme in this paper can only guarantee the privacy protection of IoT data. The privacy protection of user information involved in the transaction has not been considered. In the future, technologies such as zero-knowledge proof can be further combined to enhance privacy and security.

## Figures and Tables

**Table 1:** Comparison of blockchain systems.

Features	Bitcoin	Ethereum	Hyperledger-Fabric	IOTA
Consensus	POW	POW, POS	PBFT	Coordinator mechanism
Consensus finality	×	×	*√*	×
Blockchain forks	*√*	*√*	×	*√*
Run smart contracts	×	*√*	*√*	×
Fee less	×	×	*√*	×
Scalable	×	×	×	*√*
TX throughput	7 TPS	8-9 TPS	Thousands TPS	7-12 TPS

**Table 2:** Throughput and latency when 2G memory and one core.

Test	Send Rate	Max Latency	Min Latency	Avg Latency	Throughput
1	100 tps	0.23 s	0.01 s	0.03 s	100 tps
2	197 tps	21.95 s	0.01 s	7.43 s	160 tps
3	295 tps	31.26 s	0.10 s	17.92 s	151 tps

**Table 3:** Throughput and Latency when 4G memory and one core.

Test	Send Rate	Max Latency	Min Latency	Avg Latency	Throughput
1	100 tps	0.18 s	0.01 s	0.03 s	100 tps
2	198 tps	27.57 s	0.05 s	11.56 s	150 tps
3	296 tps	29.04 s	1.20 s	17.68 s	159 tps
4	374 tps	33.55 s	11.80 s	23.34 s	148 tps

**Table 4:** Throughput and Latency when 4G memory and two cores.

Test	Send Rate	Max Latency	Min Latency	Avg Latency	Throughput
1	100 tps	0.09 s	0.00 s	0.01 s	100 tps
2	200 tps	1.39 s	0.01 s	0.10 s	199 tps
3	299 tps	8.49 s	0.01 s	4.59 s	262 tps
4	390 tps	12.43 s	0.01 s	6.07 s	305 tps
5	496 tps	12.25 s	0.02 s	6.21 s	336 tps
6	555 tps	13.59 s	0.31 s	6.90 s	344 tps

**Table 5:** Throughput and Latency when 4G memory and four cores.

Test	Send Rate	Max Latency	Min Latency	Avg Latency	Throughput
1	100 tps	0.04 s	0.00 s	0.01 s	100 tps
2	200 tps	0.06 s	0.00 s	0.01 s	200 tps
3	399 tps	0.18 s	0.00 s	0.02 s	399 tps
4	794 tps	6.26 s	0.02 s	3.75 s	598 tps
5	1332 tps	7.34 s	1.96 s	4.77 s	645 tps
6	1503 tps	7.69 s	2.07 s	5.38 s	639 tps

**Table 6:** Peak memory usage of different client.

Node/Client	Peak Memory Usage
Tangle full node	3084 MB
Tangle light node in laptop	322.4 MB
Tangle light node in mobile development kit	23.2 MB
Consortium blockchain (Hyperledger Fabric)	1318 MB
